# Comparing ‘clinical hunch’ against clinical decision support systems (PERC rule, wells score, revised Geneva score and YEARS criteria) in the diagnosis of acute pulmonary embolism

**DOI:** 10.1186/s12890-022-02242-1

**Published:** 2022-11-21

**Authors:** Koshiar Medson, Jimmy Yu, Lovisa Liwenborg, Peter Lindholm, Eli Westerlund

**Affiliations:** 1 Department of Physiology and Pharmacology, Karolinska Institutet, 171 77 Stockholm, Sweden; 2 Department of Imaging and Physiology, Cardiothoracic Section, Karolinska University Hospital, 171 76 Stockholm, Sweden; 3 Departments of Molecular Medicine and Surgery, Karolinska Institutet, 182 88 Stockholm, Sweden; 4 Department of Emergency Medicine, University of California San Diego, San Diego, USA; 5 Department of Clinical Sciences, Karolinska Institutet, 182 88 Stockholm, Sweden; 6 Department of Internal Medicine, Danderyd Hospital, Stockholm, Sweden

**Keywords:** Pulmonary embolism, Clinical decision support systems, CTPA, Diagnostics, Wells score, PERC rule, Revised Geneva score, YEARS criteria

## Abstract

**Background:**

Pulmonary embolism (PE) is a common and potentially life-threatening condition. Since it is considered a ‘do not miss’ diagnosis, PE tends to be over-investigated beyond the evidence-based clinical decision support systems (CDSS), which in turn subjects patients to unnecessary radiation and contrast agent exposure with no apparent benefits in terms of outcome.

The purpose of this study was to evaluate the yield of ‘clinical hunch’ (gestalt) and four CDSS: the PERC Rule, Wells score, revised Geneva score, and Years criteria.

**Methods:**

A review was conducted on the Electronic Medical Records (EMR) of 1566 patients from the Emergency Department at a tertiary teaching hospital who underwent CTPA from the 1st of January 2018 to the 31st of December 2019. The scores for the four CDSS were calculated retrospectively from the EMR data. We considered that a CTPA had been ordered on a clinical hunch when there was no mention of CDSS in the EMR, and no D-dimer test. A bypass of CDSS was confirmed when any step of the diagnostic algorithms was not followed.

**Results:**

Of the total 1566 patients who underwent CTPA, 265 (17%) were positive for PE. The diagnosis yield from the five decision groups (clinical hunch and four CDSS) was as follows—clinical hunch, 15%; PERC rule, 18% (6% when bypassed); Wells score, 19% (11% when bypassed); revised Geneva score, 26% (13% when bypassed); and YEARS criteria, 18% (6% when bypassed).

**Conclusion:**

Clinicians should trust the evidence-based clinical decision support systems in line with the international guidelines to diagnose PE.

**Supplementary Information:**

The online version contains supplementary material available at 10.1186/s12890-022-02242-1.

## Background

Pulmonary embolism (PE) is considered one of the ‘do-not-miss’ diagnoses in emergency departments (ED). It has a very unspecific symptom spectrum, including chest pain and dyspnea, that overlaps with many other conditions. The long list of predisposing risk factors makes the diagnosis of PE difficult and complicated [1, 2].

PE is the third most common cause of death among cardiovascular diseases after myocardial infarction and stroke, with approximately 300,000 deaths in Europe annually [3, 4].

The mortality rate is 15% within 3 months and 20% in the first year after diagnosis [2].

Imaging has become the mainstay for diagnosing PE and is used often to investigate a ‘clinical hunch’. Computed Tomography Pulmonary Angiography (CTPA) is the imaging modality of choice, due to its high sensitivity and specificity, and being readily available in most hospitals. However, CTPA has its drawbacks with the two main concerns being radiation exposure and the iodinated contrast medium [5, 6].

Data show that the number of CTPA performed has increased in the last two decades and that there has also been an increase in the number of diagnosed PE cases. Interestingly, there has not been any significant mortality rate change during the same period [7–9].There is no established expected general PE diagnosis yield from the CTPA performed, but a value of 10% or above is generally accepted in the US, although this value differs geographically as the expectation in Europe is about 20–30% [10].

Several Clinical Decision Support Systems (CDSS) have been developed to give clinicians the tools needed to assess the probability of, and the ability to exclude PE, without performing CTPA. The first CDSS was the Wells score, which was introduced in 2001, followed by the PERC rule in 2004, the Revised Geneva score in 2006, and the YEARS Criteria in 2017 [11–18].

The use of CDSS would help to reduce the number of CTPAs made, and to increase the PE diagnosis yield. In turn, this would lead to lower radiation and contrast medium exposure, while lowering the cost and waiting time in the emergency department. The present study explores the differences in yield between the available CDSS versus clinical hunch and assesses the yield when the CDSS are [7] bypassed [19]

## Methods

The study was performed according to the Declaration of Helsinki and approved by the local Ethics Committee.

We reviewed the Electronic Medical Record (EMR) of all patients aged ≥ 18 years who were suspected of having PE and admitted to the Emergency Department (ED) at a tertiary teaching hospital then underwent CTPA. The inclusion period was 1st of January 2018 to 31st of December 2019. Exclusion criteria were: less than six months between two CTPA for the same patient, pregnancy or if necessary data to calculate the CDSS scores were missing in the EMR. Pregnant patients were excluded to simplify the comparison between algorithms, as the only CDSS validated for pregnancy is the modified YEARS criteria.

We extracted the necessary data from the EMR for the CDSS algorithms and then calculated the risk of PE. The four CDSS algorithms used were PERC rule, Wells score, Revised Geneva score and Years Criteria.

An algorithm was classified as overridden or bypassed when a CTPA was ordered in patients excluded by the CDSS, or in patients in the low- or intermediate-risk group who had a negative or no D-dimer, although the CDSS algorithm required it. The classification that a CTPA had been ordered based on a clinical hunch was given when there was no mention of any CDSS in the EMR, and no D-dimer was taken.

All the test characteristics of the following four CDSS are shown in Additional file 1.

The PERC rule is used to exclude PE without using D-dimer in low-risk patients. If any of the criteria are positive, PE cannot be excluded [13, 17].

The Wells score has a two- and a three-tier model. We chose to use the two-tier model, which provides a PE unlikely (score ≤ 4) and PE likely (score ≥ 5) [12, 18].

Due to the criteria “PE is #1 diagnosis or equally likely”, we calculated two Wells score versions. In version A, we gave all the patients 3 points, and in version B, we only gave 3 points to the patient if PE was in the differential diagnosis list and/or there was any mention of CDSS in the EMR.

The revised Geneva score divides the patients into a low-, intermediate- and high-risk groups. In the low-risk group (score 0–3), the workup can stop, In the intermediate group (score 4–10), the next step is D-dimer, and in the high-risk group (score 11 +), the next step is imaging [14, 16].

The YEARS criteria use three of the Wells criteria but also incorporates D-dimer in the algorithm for all patients. A combination of D-dimer level and clinical signs and symptoms will either exclude PE or suggest imaging unless the patient is pregnant, where the algorithm takes a slightly different route [11, 15].

All the criteria in these four CDS are described in Additional file 1.

As with the Wells score due to the criteria “PE most likely diagnosis”, we calculated two versions. In version A, we gave all the patients 1 point, and in version B, we only gave the patients 1 point if PE was in the differential diagnosis list and/or there was any mention of CDSS in the EMR.

All statistical analysis was calculated with MedCalc online statistical software and Microsoft Excel.

## Results

**Fig. 1:**
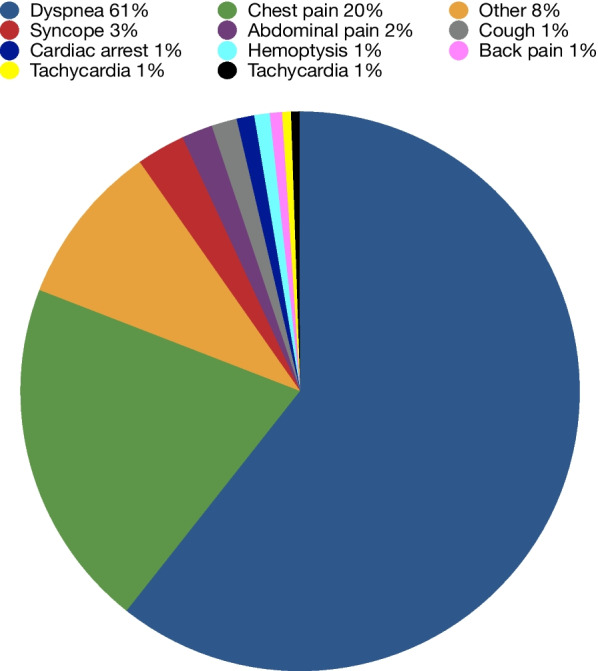
Reason for ED visits

There were 1,566 patients included in this study; 265 had positive (17%), and 1,301 (83%) had negative CTPAs. The cohort included 840 female and 726 male patients, aged between 18 and 103 years old, with a median age of 64. The main reason for a patient visiting the ED was dyspnea (*n* = 950), chest pain (*n* = 317), syncope (*n* = 44), abdominal pain (*n* = 28), cough (*n* = 23), cardiac arrest (*n* = 16), hemoptysis (*n* = 14), back pain (*n* = 11), tachycardia (*n* = 8) and other (*n* = 143) as shown in Fig. 1.

The distribution levels of the emboli were as followed: central/lobar (*n* = 137), segmental (*n* = 73) and subsegmental (*n* = 55). In 257 cases, an alternative diagnosis could entirely or partially explain the patient’s symptoms, such as pleural or pericardial effusion, pneumonia, chronic obstructive pulmonary disease exacerbation, suspected metastasis, primary malignancy, or bronchiolitis. D-dimer was tested for in 751 patients (48%). There was mention of CDSS in the EMR only in 74 (5%) out of the 1,566 patients.

The clinical hunch group contained 786 patients who fulfilled the inclusion criteria, with 118 positive (15%) and 668 negative (85%) cases, resulting in a yield of 15%.

The PERC rule resulted in 1421 patients in the not-excluded group, of whom 256 had a positive and 1165 a negative CTPA, resulting in a yield of 18%. Out of the 145 patients excluded, 9 had a positive and 136 a negative CTPA, resulting in a yield of 7%. When overriding the PERC rule, the yield was lowered from 18 to 6%.

The wells score version A resulted in 844 patients in the high-risk group, of whom 177 had a positive, and 667 a negative CTPA. Of the 722 patients in the low-risk group, 318 had a positive D-dimer, of whom 45 had a positive and 273 a negative CTPA resulting in a yield of 19%. Of the remaining 404 patients who had a negative or no D-dimer, 43 had a positive and 361 a negative CTPA, resulting in a yield of 11%. When overriding the Wells score, the yield was lowered from 19 to 11%.

The Wells score version B resulted in 147 patients in the high-risk group, of whom 42 had a positive, and 105 a negative CTPA. Out of the 1419 patients in the low-risk group, 593 had a positive D-dimer, of whom 112 had a positive, and 481 a negative CTPA resulting in a yield of 21%. Of the remaining 826 patients who had a negative or no D-dimer, 111 had a positive and 715 a negative CTPA, resulting in a yield of 13%. When overriding the Wells score, the yield was lowered from 21% to 13.

The Revised Geneva score resulted in 113 patients in the high-risk group, of whom 39 had a positive, and 74 a negative CTPA. Of the 970 patients in the intermediate-risk group, 378 had a positive D-dimer, of whom 88 had a positive, and 290 a negative CTPA, resulting in a yield of 26%. Of the remaining 1075 patients who had a negative, or no D-dimer, or were in the low-risk group, 93 had a positive, and 982 a negative CTPA, resulting in a yield of 13%. When overriding the revised Geneva score, the yield was lowered from 26 to 13%.

The Years criteria Version A group contained 751 patients who fulfilled the inclusion criteria by having the D-dimer tested. This resulted in 657 patients in the not-excluded group, of whom 121 had a positive, and 519 a negative CTPA. Of 94 patients in the excluded group, 6 had a positive and 88 a negative CTPA resulting in a yield of 18%. When overriding the YEARS criteria version A, the yield was lowered from 18 to 6%.

**Fig. 2:**
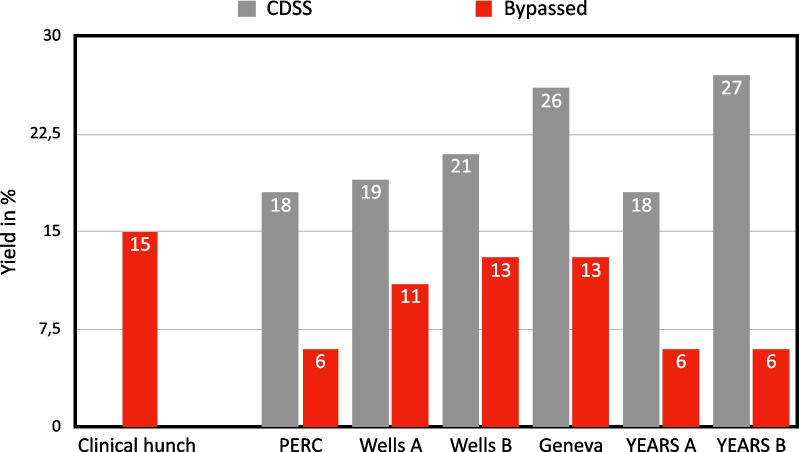
Yield differences between CDSS and when bypassed

**Fig. 3:**
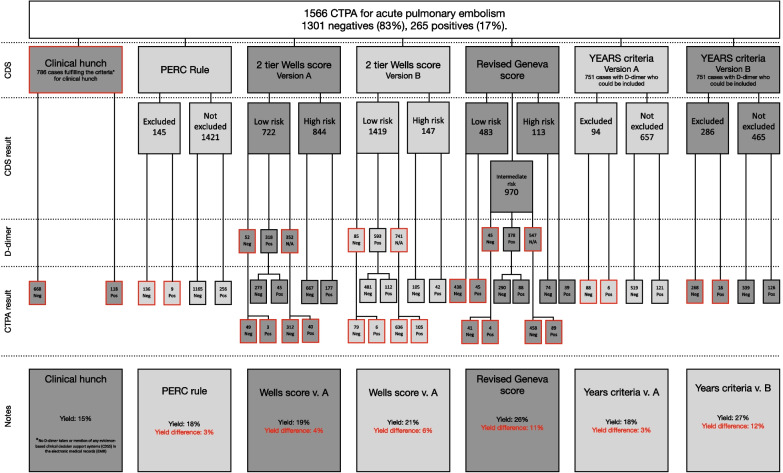
Flowchart for the clinical decision support systems (CDSS)

The Years criteria Version B resulted in 465 patients in the not-excluded group, of whom 126 had a positive, and 339 a negative CTPA. Of the 286 patients in the excluded group, 18 had a positive and 268 a negative CTPA resulting in a yield of 27%. The remaining 904 patients had no D-dimer and therefore could not be included. When overriding the YEARS criteria version B, the yield was lowered from 27 to 6%. Figures 2 and 3 visualize the results and the yield when bypassed.

A total of 94 patients had a negative d-dimer. Three had PE at the subsegmental level. The main complaint was dyspnoea (*n* = 2), haemoptysis (*n* = 1).

In 55 cases, the CTPA was ordered after the results of the d-dimer were available. The average time between the d-dimer result and the CTPA order was 3 h: 30 min (minimum 4 min and maximum 23 h: 42 min).

In 17 cases, the CTPA was ordered before the d-dimer with an average time difference of 42 min. (minimum 1 min and maximum 3 h: 54 min).

In 22 cases, the CTPA was ordered after the d-dimer with an average time between the orders of 24 min. (minimum 1 min, maximum 57 min).

## Discussion

As the present study found that CDSS gave a higher yield compared to clinical hunch, clinicians should trust the evidence-based clinical decision support systems in line with the international guidelines to diagnose PE.

Several other studies have shown that the CTPA PE yield is lower when the CDSS is bypassed. In our study, the decrease in yield ranged from 8 to 21%. The latest recommendations from the European Society of Cardiology (ESC) (2019) state that CDSS should be used as a decision tool in the PE diagnostic process [20]. The question is, why do clinicians bypass the CDSS algorithms?

In order to address this question, we started by comparing the bypassed and non-bypassed group to find parameters that might cause the clinician to sidestep the system. However, no definitive findings, including patterns of physical signs and symptoms, or laboratory values that might cause bypass, were identified. Some studies have noted that some clinician-perceived risk factors such as malignancy (last treatment < 1 year prior), travel ≥ 4 h, and thrombophilia are not included in any of the CDSS [21]. These omissions might explain a few of the cases in our study but not all. In many cases, a CTPA was ordered for patients in a low-risk group who also had a negative D-dimer.

The following options may explain why a CTPA is ordered:

- Clinicians do not want to miss PE, despite the radiation and contrast medium risk, not to mention the cost and time involved.
- CTPA has become the go-to examination in patients with these symptoms because it might give other explanations if PE is negative.
- Awareness or ease of use/availability of CDSS could be another reason.
- Logistical issues and/or patient pressure.

Any combination of these four factors could be a reason for clinicians to include PE in their differential diagnosis, and thus order a CTPA more often than necessary.

There are an increasing number of CTPAs being made to diagnose PE (and an increase in CT use in general), but not a proportional increase in diagnosis yield. [7–9] This increase in use has also lead to the diagnosis of smaller emboli at the subsegmental level, which may not be clinically relevant; the decision on whether to treat subsegmental emboli is still being debated, considering there is a 7% risk of major bleeding associated with anticoagulation therapy. [22–24] In our study, 21% of the diagnosed PE were subsegmental.

There were also 257 cases (17.6%) in our study who received an alternative diagnosis that could entirely or partially explain the patient’s symptoms, but we believe many of these diagnoses, like pneumonia or pleural effusion, does not justify or necessitate the use of CT for diagnosis [8]. Incidental findings may lead to unnecessary investigations with no apparent benefit for the patient [25].

There have been attempts to incorporate the CDSS in a computerized order system. These system integrations have shown an increase in adherence to the guidelines when ordering imaging diagnostics, which increases the yield [26–28]. Integration of these systems could also increase the awareness of CDSS. Integration of the CDSS in the radiology order system is the next logical step for further evaluation of the ordering behavior at our hospital.

Overordering of CTPAs could also be a purely logistical issue. Emergency rooms are overcrowded, and the waiting times are generally long. The addition of PE to the list of differential diagnoses when ordering a chest CT will raise the exam to a higher priority level, meaning that instead of the usual maximum of three hours for a regular chest CT, it will be performed within one hour. Thus, the waiting time for the patient in the ED and the handling time for the doctors will be shorter, but the request will put an unsustainable pressure on the radiology department. Patients may also pressure doctors in the ED for a more extensive diagnostic procedure than medically necessary and justified, often following the patient researching their condition on the internet and demanding a particular diagnostic procedure.

Regarding the limitations**,** this study was conducted retrospectively and relied on the information extracted from the EMR. Although we could extract the necessary information to calculate the CDSS, we lacked the clinical context and any information that the treating clinician did not record. We tried to compensate for at least part of the latter by calculating two versions of the Wells score and the YEARS criteria. In addition, the availability of diagnostic resources at a tertiary teaching hospital is likely to be increased over that of smaller hospitals, and thus the ordering behavior of doctors is likely to vary with the availability of diagnostic resources. Therefore, this study might not be representative of smaller hospitals or those in more remote areas. Lastly, the present study is a single center.

## Conclusion

The CDSS have been developed to help clinicians make better decisions regarding the diagnosis of PE. They are used to increase the PE diagnostic yield of CTPA, thereby avoiding exposure to unnecessary radiation, contrast medium and ultimately, reducing patient anxiety. As radiologists are not infallible, some emboli cases will always be missed even if all patients entering the ED are scanned. The CDSS do have room for improvement, with new and revised versions being developed. We believe that clinicians should trust and follow the CDSS as recommended by guidelines to give the most benefit to their patients.

## Supplementary Information

**Additional file 1**. The criteria for the four available Clinical Decision Support systems and how pulmonary embolism risk is calculated using each.

## Data Availability

The datasets during and/or analyzed during the current study available from the corresponding author on reasonable request.

## Abbreviations

PE: Pulmonary embolism
CDSS: Clinical decision support systems
EMR: Electronic medical records
CTPA: Computed tomography pulmonary angiography
CT: Computed tomography
ED: Emergency department
ESC: European society of cardiology
