# Supplementary material for: Comparing ‘clinical hunch’ against clinical decision support systems (PERC rule, wells score, revised Geneva score and YEARS criteria) in the diagnosis of acute pulmonary embolism
Source: BMC Pulm Med. 2022 Nov 21;22:432. doi: 10.1186/s12890-022-02242-1 (PMC9682736; doi:10.1186/s12890-022-02242-1)
Supplement: Supplementary file 1 — Additional file 1. The criteria for the four available Clinical Decision Support systems and how pulmonary embolism risk is calculated using each. [file 12890_2022_2242_MOESM1_ESM.docx]

**PERC rule:**

| Age ≥50 | 1 |
| --- | --- |
| HR ≥100 | 1 |
| O₂ sat <95% | 1 |
| Unilateral leg swelling | 1 |
| Hemoptysis | 1 |
| Recent surgery or trauma  Surgery or trauma ≤4 weeks ago requiring treatment with general anesthesia | 1 |
| Prior PE or DVT | 1 |
| Hormone use  Oral contraceptives, hormone replacement or estrogenic hormones use in males or female patients | 1 |

If any of the criteria are positive, PE can not be ruled out.

**Wells criteria:**

| Clinical signs and symptoms of DVT | 3 |
| --- | --- |
| PE is the #1 diagnosis or equally likely | 3 |
| Heart rate > 100 | 1.5 |
| Immobilization at least 3 days OR surgery in the previous 4 weeks | 1.5 |
| Previous, objectively diagnosed PE or DVT | 1.5 |
| Hemoptysis | 1 |
| Malignancy w/ treatment within 6 months or palliative | 1 |

Three tier:

Low risk < 2 points

Intermediate risk 2-6 points

High risk > 6 points

Two tier:

PE unlikely 0-4 points

PE likely > 4 points

**Revised Geneva score:**

| Age > 65 | 1 |
| --- | --- |
| Previous DVT or PE | 3 |
| Surgery (under general anesthesia) or lower limb fracture in past month | 2 |
| Active malignant condition  Solid or hematologic malignant condition, currently active or considered cured < 1 year | 2 |
| Unilateral lower limb pain | 3 |
| Hemoptysis | 2 |
| Heart rate <75 | 0 |
| Heart rate 75 - 94 | 3 |
| Heart rate ≥ 95 | 5 |
| Pain on lower limb palpation and unilateral edema | 4 |

Low risk 0 – 3 points

Intermediate risk 4 – 10 points

High risk ≥ 11 points

**YEARS criteria:**

| Clinical signs of DVT | 1 |
| --- | --- |
| Hemoptysis | 1 |
| PE most likely diagnosis | 1 |
| D-dimer ≥500 | Yes/No |
| D-dimer ≥1,000 | Yes/No |

PE excluded when

0 point and D-dimer <1,000 or ≥1 point and D-dimer <500

PE not excluded, order CTPA

0 point and D-dimer ≥1,000 or ≥1 point and D-dimer ≥500
